# Confronting Upside-Down Video Assisted Thoracic Surgery for Posterior Mediastinal Müllerian Duct Cysts

**DOI:** 10.7759/cureus.101997

**Published:** 2026-01-21

**Authors:** Momoka Harada, Tomonari Oki, Shuhei Iizuka, Yoshiro Otsuki, Toru Nakamura

**Affiliations:** 1 Department of General Thoracic Surgery, Seirei Hamamatsu General Hospital, Hamamatsu, JPN; 2 Department of Pathology, Seirei Hamamatsu General Hospital, Hamamatsu, JPN

**Keywords:** mediastinal neoplasms, müllerian ducts, pathology, thoracic surgery, vats, video-assisted, video-assisted thoracoscopic surgery (vats)

## Abstract

A Müllerian duct cyst is a rare epithelial-lined cyst originating from remnants of the Müllerian (paramesonephric) duct. While these cysts are typically found along the lateral or posterior vaginal wall in females or near the prostate in males, posterior mediastinal occurrence is exceedingly rare. Accurate preoperative diagnosis is challenging, as imaging findings often resemble those of more common posterior mediastinal lesions such as neurogenic tumors or bronchogenic cysts. Surgical resection is usually required for definitive diagnosis and treatment. Video-assisted thoracic surgery (VATS) via the confronting upside-down monitor setting is a novel approach that offers improved visualization and ergonomics, but has not previously been reported for resection of a posterior mediastinal Müllerian duct cyst.

A 44-year-old woman was referred following the incidental detection of a right posterior mediastinal mass on chest radiography. She was asymptomatic, with normal laboratory results including tumor markers. Contrast-enhanced computed tomography (CECT) revealed a well-defined, non-enhancing mass adjacent to the right T5-T6 vertebral bodies, measuring 3.3 × 1.8 × 2.8 cm. Magnetic resonance imaging (MRI) showed a cystic lesion with high T2 signal intensity. A neurogenic tumor or bronchogenic cyst was suspected, and surgical resection was performed using a four-port VATS approach with the confronting upside-down monitor setting. Intraoperatively, a smooth-surfaced cyst originating from the T5 vertebral body was identified and resected en bloc with the adherent fifth intercostal vein. Histopathology revealed a cyst lined by cuboidal epithelium, positive for Claudin4, estrogen receptor, Wilms' Tumor 1 (WT1), and Paired box gene 8 (PAX8), and negative for Calretinin, findings consistent with a Müllerian duct cyst. The patient’s postoperative course was uneventful, and she was discharged on postoperative day two.

Posterior mediastinal Müllerian duct cysts, though rare, should be considered in the differential diagnosis of mediastinal cysts in perimenopausal women. Definitive diagnosis requires histopathological and immunohistochemical evaluation. VATS via the confronting upside-down monitor setting provides direct visualization, minimal instrument interference, and improved spatial orientation, making it a valuable surgical option, particularly for lesions in close proximity to the intercostal vessels or the vertebral column. This case represents the first reported resection of a posterior mediastinal Müllerian duct cyst using this approach.

## Introduction

A Müllerian duct cyst is an epithelial-lined cyst derived from the Müllerian (paramesonephric) duct [1], which forms during the embryonic period. These cysts are more commonly observed in adult women, particularly around the perimenopausal period. Although one hypothesis suggests that remnants of Müllerian duct-derived tissue from the embryonic period may undergo cystic transformation in response to hormonal stimulation, the exact etiology remains unclear [2,3].

In female subjects, the Müllerian duct differentiates into the fallopian tubes, uterus, and upper vagina, while in male subjects, it typically regresses, persisting as the prostatic utricle. Consequently, Müllerian duct cysts typically occur along the lateral or posterior wall of the vagina in female subjects and in the pelvic cavity, particularly near the prostate, in male subjects [4].

On rare occasions, they may arise in the posterior mediastinum [5]. Posterior mediastinal Müllerian duct cysts are typically asymptomatic; however, they may present with a variety of symptoms, including discomfort, chest pain, or dyspnea [6]. In such cases, differential diagnosis is required to distinguish them from the more common posterior mediastinal lesions such as neurogenic tumors and bronchogenic cysts. Due to the challenges of accurate imaging-based diagnosis, surgical resection is often necessary, for both definitive diagnosis and treatment [7-9]. 

Previously reported surgical approaches include thoracotomy, conventional looking-up video-assisted thoracic surgery (VATS), and robot-assisted thoracic surgery (RATS) [10,11]. 

Recently, the confronting upside-down monitor setting has been introduced as a novel modification of VATS [12], primarily developed to improve ergonomic access and visualization during lung cancer surgery. Although this approach has gradually been applied to selected mediastinal tumors, its use remains limited. 

To our knowledge, this is the first report describing the resection of a posterior mediastinal Müllerian duct cyst using the confronting upside-down VATS approach.

## Case presentation

**Figure 1:**
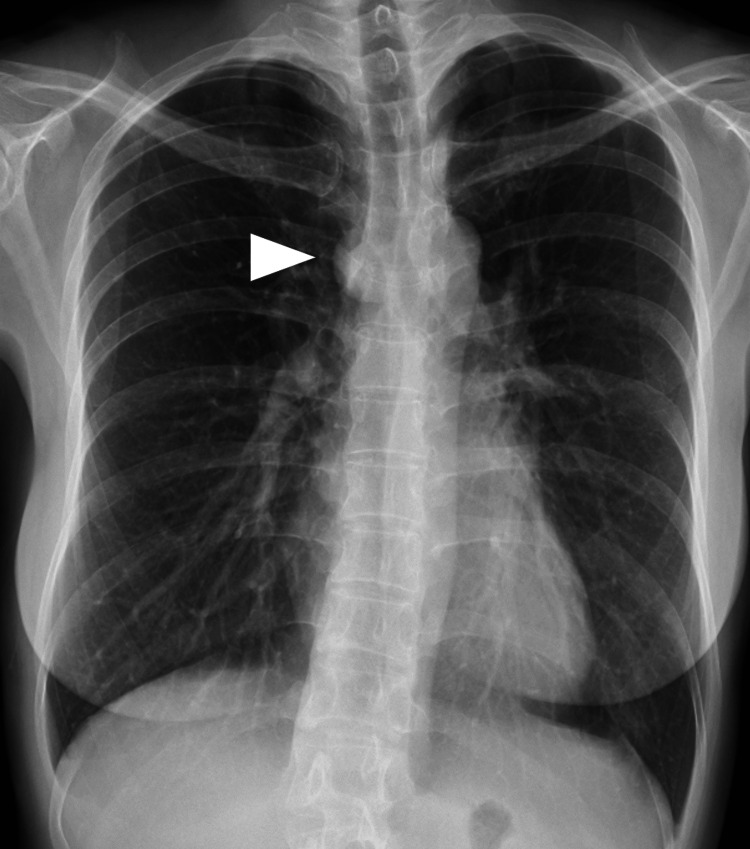
Chest radiography A chest radiograph showing a well-defined nodule in the mediastinum (arrowhead).

A 44-year-old woman was referred to our department after an abnormal shadow was detected on chest radiography during a routine health screening (Figure 1).

She had no relevant medical history, was not on any medications, and had never smoked. Her family history was notable for her father’s diagnosis of prostate cancer. We also considered the possibility of metastatic cancer, germinal tumor or malignant lymphoma and therefore performed a series of diagnostic tests, including tumor marker evaluation. Laboratory tests, including serum levels of human chorionic gonadotropin (HCG), carcinoembryonic antigen (CEA), alpha-fetoprotein (AFP), cancer antigen 125 (CA125), and soluble interleukin (IL)-2 receptor, were all within normal limits (Table 1).

**Table 1:** Summary of the patient’s laboratory data

Laboratory test	Patient value	Reference range	Units
Human chorionic gonadotropin (HCG)	<2.30	<5.0	mIU/mL
Carcinoembryonic antigen (CEA)	<1.73	0-5	ng/mL
Alpha-fetoprotein (AFP)	2.81	0-8.78	ng/mL
Cancer antigen 125 (CA125)	14.2	0-35	U/mL
Soluble IL-2 receptor (sIL-2R)	289	157-474	U/mL

**Figure 2:**
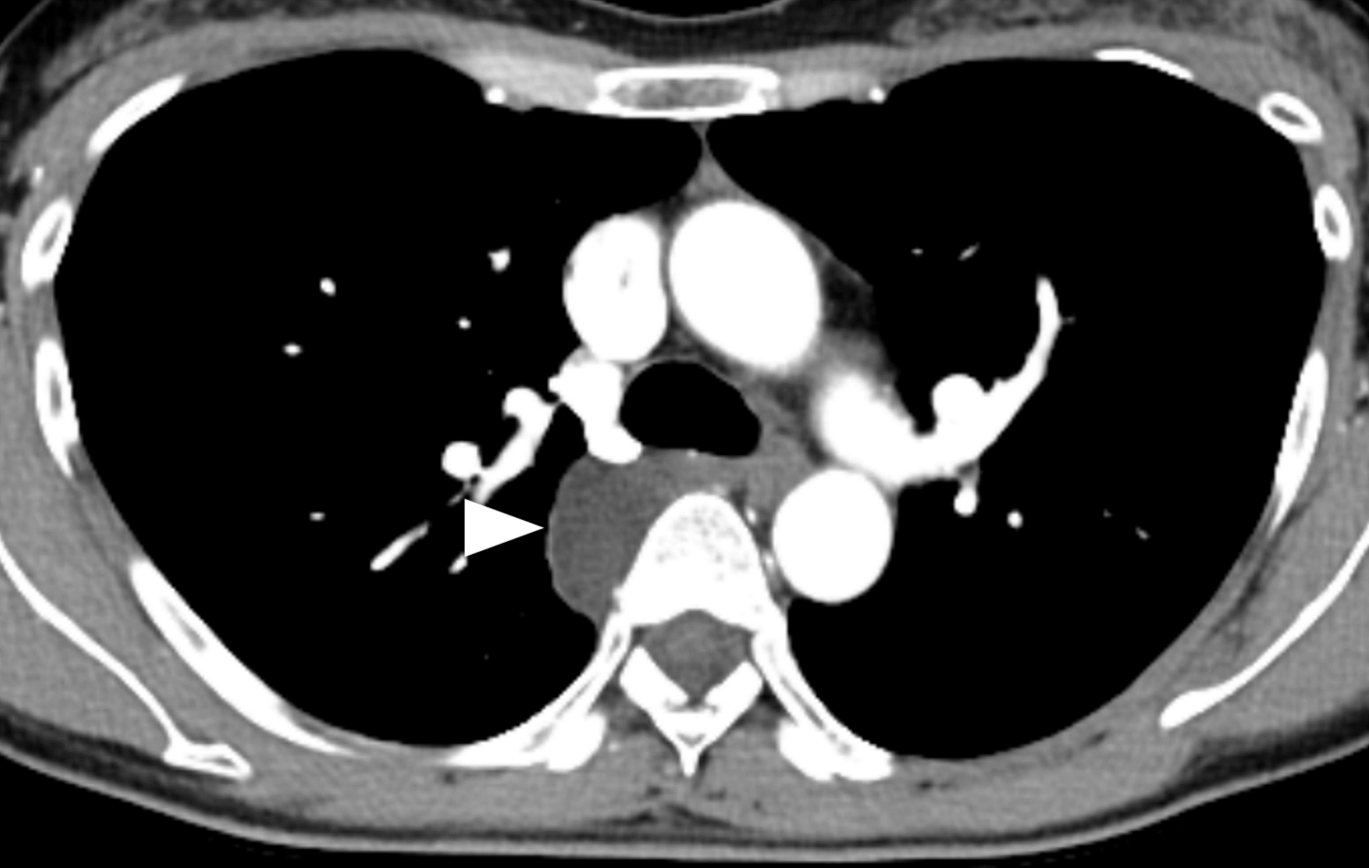
Contrast-enhanced computed tomography (CECT) Contrast-enhanced axial CT image showing a well-defined, non-enhancing lesion along the right side of the vertebral body (arrowhead).

No significant clinical findings were noted. Contrast-enhanced computed tomography (CECT) revealed a well-defined, non-enhancing mass adjacent to the right side of the T5 and T6 vertebral bodies, measuring 3.3 cm × 1.8 cm × 2.8 cm (Figure 2).

**Figure 3:**
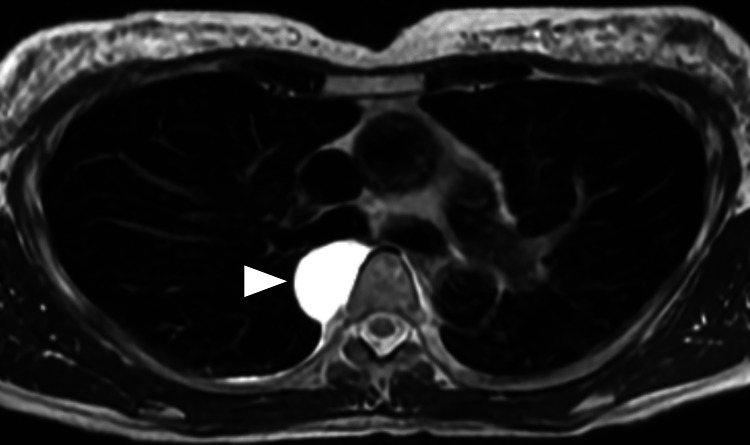
Chest magnetic resonance imaging (MRI) T2-weighted MRI showing a hyperintense lesion suggestive of a cystic nature (arrowhead).

Magnetic resonance imaging (MRI) showed a cystic lesion at the same site, showing high signal intensity on T2-weighted images (Figure 3).

No additional abnormalities or lesions were identified elsewhere. Based on the imaging findings, a neurogenic tumor or foregut cysts was suspected, and surgical resection was planned.

**Figure 4:**
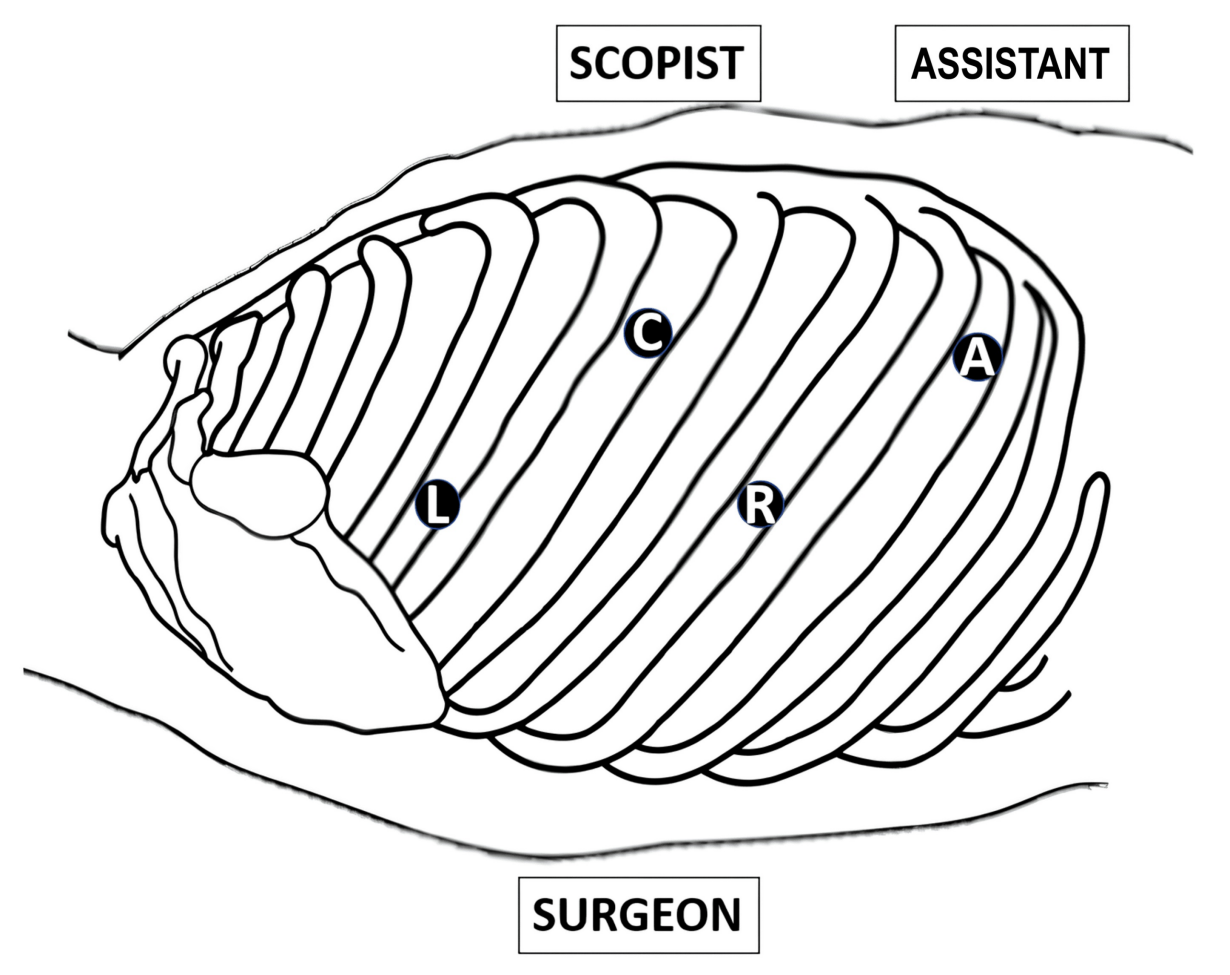
Port settings A right-hand port for the surgeon (R) placed in the seventh intercostal space (ICS) along the posterior axillary line; a 7-mm left-hand port for the surgeon (L) in the fourth ICS along the posterior axillary line; an 11-mm camera port (C) in the fifth ICS along the anterior axillary line; and an assistant port (A) in the eighth ICS along the anterior axillary line.

The patient was placed in the left lateral decubitus position. A four-port VATS approach with the confronting upside-down monitor setting was employed (Figure 4).

A wound retractor was inserted at the posterior axillary line of the seventh intercostal space, an 11-mm port at the anterior axillary line of the fifth intercostal space, a 7-mm port at the posterior axillary line of the fourth intercostal space, and another wound retractor at the anterior axillary line of the eighth intercostal space.

**Figure 5:**
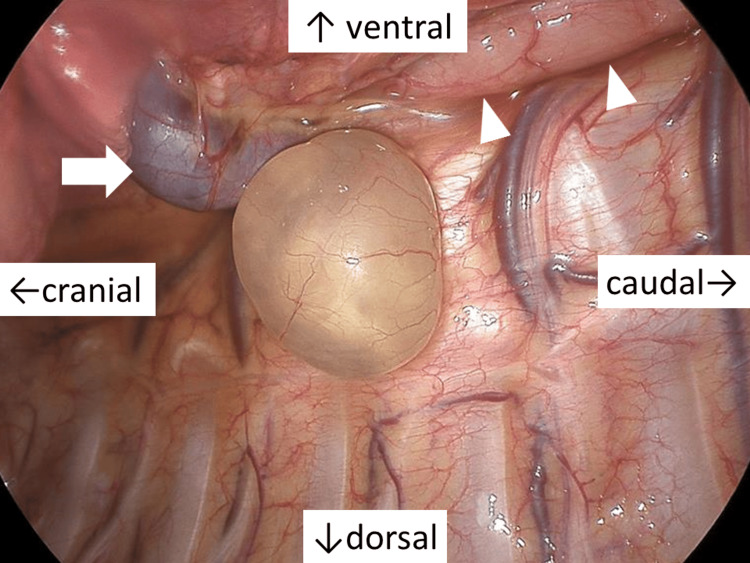
Operative view A smooth-surfaced cystic lesion with serous contents, broadly arising from the body of the fifth thoracic vertebra. On the ventral side of the cyst, the azygos vein (arrow) and esophagus (arrow heads) are identified in cranio-caudal order.

Thoracoscopic findings revealed a broad-based, smooth-surfaced cystic lesion containing serous fluid originating from the thoracic five (T5) vertebral body (Figure 5).

The dorsal surface of the cyst was sharply dissected from the parietal pleura, progressing around approximately half its perimeter. During dissection from the paravertebral attachment, the cyst wall was inadvertently ruptured, resulting in leakage of serous fluid, which was aspirated as needed. The cyst was closely associated with the fifth intercostal vein, which was ligated proximally and distally with 2-0 silk sutures and resected en bloc with the cyst.

Following copious irrigation, a 20-Fr single-lumen drain was placed dorsally through the port site at the anterior axillary line of the eighth intercostal space, and the surgical wounds were closed.

**Figure 6:**
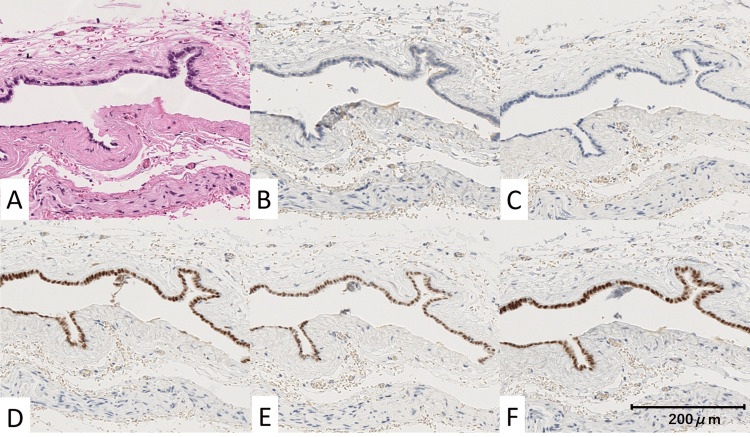
Immunohistochemical staining findings A: Hematoxylin–eosin staining showing a cyst lined by a single layer of cuboidal epithelium (×20); B: Positive Claudin-4 staining in the epithelial cells (×20); C: Negative calretinin staining in the epithelial cells (×20); D: Positive estrogen receptor (ER) staining in the epithelial cells (×20); E: Positive Wilms' tumor 1 (WT1) staining in the epithelial cells (×20); F: Positive paired box gene 8 (PAX8) staining in the epithelial cells (×20).

Histopathological examination revealed a cyst lined by a single layer of cuboidal epithelium. Immunohistochemical staining showed positivity for Claudin4, estrogen receptor (ER), Wilms' tumor 1 (WT1), and paired box gene 8 (PAX8), and negative for Calretinin, consistent with a diagnosis of a Müllerian duct cyst (Figures 6).

The postoperative course was uneventful, and the patient was discharged on postoperative day two.

## Discussion

Posterior mediastinal tumors comprise a heterogeneous group of pathologies, the majority of which, approximately 75-95%, are of neurogenic origin [13]. Other lesions arising in this region include bronchogenic and foregut-derived cysts, lymphomas, lymphadenopathy, and mesenchymal tumors such as liposarcomas and leiomyomas. Clinically, Müllerian cysts in female patients, also known as paraventricular cysts, are extremely rare. Mediastinal cysts composed of Müllerian epithelium are a recently recognized entity, accounting for less than 1% of all cystic lesions in this anatomical compartment [6]. Most reported cases have involved women in the perimenopausal period. Although the precise pathogenesis remains uncertain, the presence of ectopic Müllerian tissue has been hypothesized, with proposed mechanisms including embryonic remnants or coelomic epithelial metaplasia. In addition, associated risk factors include obesity and gynecologic history, such as hormone replacement therapy. In women who are obese, elevated estrogen levels are attributed to increased aromatase activity in adipose tissue [14]. Radiological imaging alone is often insufficient to differentiate Müllerian cysts from more common posterior mediastinal lesions such as neurogenic tumors or bronchogenic cysts [15]. Histologically, the cyst wall is typically lined by a single layer of columnar epithelial cells. Immunohistochemical staining usually demonstrates positivity for ER and progesterone receptor (PgR), findings that are characteristic of Müllerian differentiation and supportive of the diagnosis [16]. Given their rarity, Müllerian cysts are not commonly considered in the differential diagnosis of posterior mediastinal cysts. Nevertheless, clinicians should maintain a high index of suspicion, particularly in premenopausal or perimenopausal women, as accurate diagnosis requires a thorough pathological evaluation, including immunohistochemical analysis, to distinguish these lesions from more prevalent entities. Furthermore, the provision of relevant clinical information, such as the patient’s age and menopausal status, is essential to facilitate accurate histopathological interpretation by pathologists.

Various surgical approaches for posterior mediastinal Müllerian cysts have been reported, including thoracotomy, VATS, and RATS. Among these, VATS with the look-up setting is most commonly employed. In this method, the camera port is typically placed in the lower intercostal spaces (seventh or eighth), and the monitor is positioned cephalad to the patient, providing a cranial-to-caudal view of the thoracic cavity. However, this approach presents several limitations. The instrument tips can easily deviate from the visual axis, particularly near the cranial aspect of the lesion, resulting in blind spots. Furthermore, the caudal insertion of the scope often results in a mirror image when instruments are advanced along the visual axis. This may hinder precise and coordinated manipulation [17].

**Figure 7:**
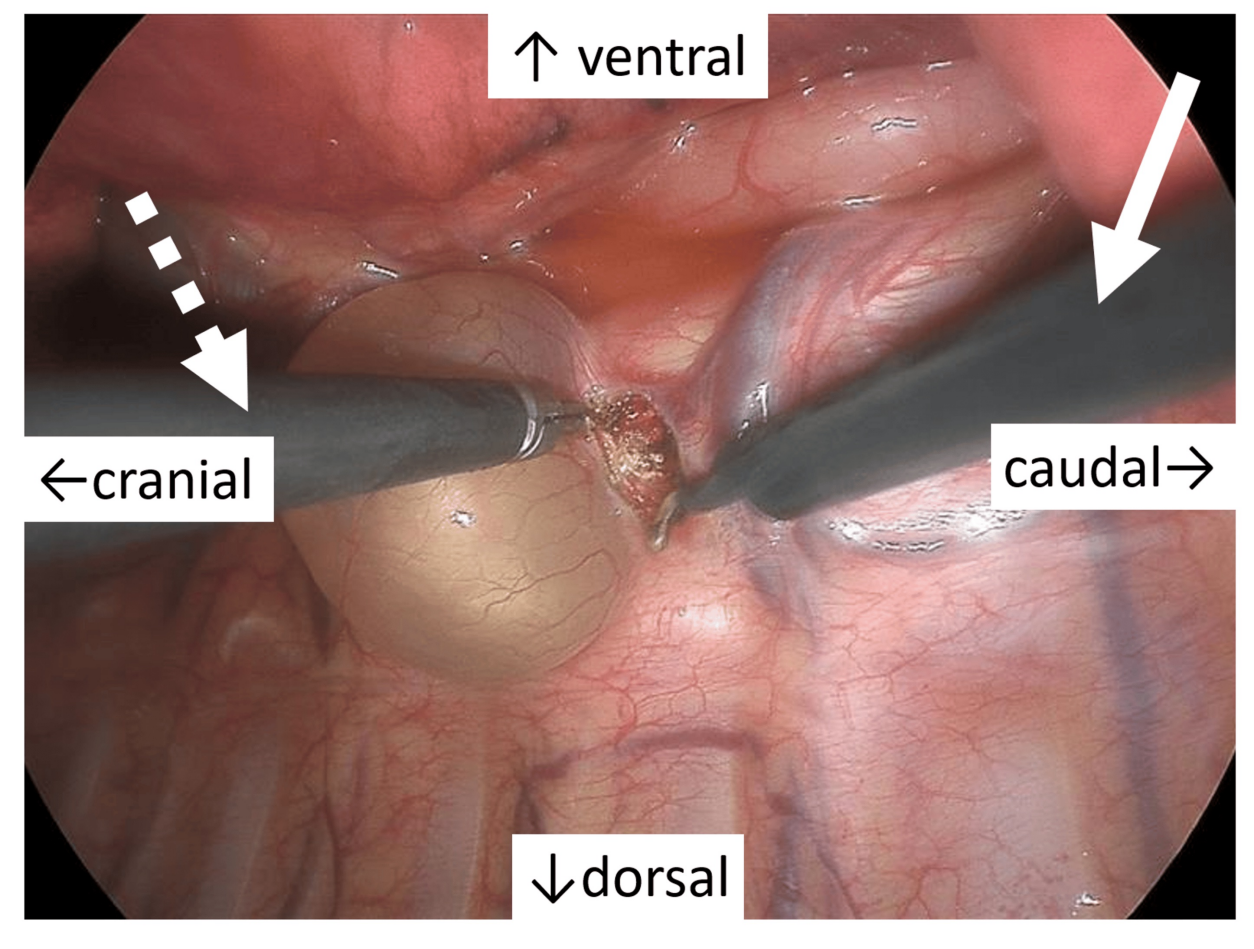
Operative view Instruments are inserted caudally (right hand: arrow) and cranially (left hand: dotted arrow), enabling operative maneuverability comparable to that of open thoracotomy.

To address these limitations, VATS using the confronting upside-down monitor setting has recently been introduced, primarily in pulmonary lobectomy [12]. In this technique, the thoracoscope is inserted through higher intercostal spaces (typically the third to fifth), and the assistant’s monitor is rotated 180°, allowing both the surgeon and assistant to share a direct, non-mirrored field of view. This configuration replicates the intuitive spatial orientation of open thoracotomy, reduces instrument interference, and facilitates precise and coordinated surgical maneuvers (Figure 7).

In the present case, the cyst was firmly adherent to the intercostal vein and required en bloc resection. This approach provided a direct view of the lesion and adjacent vessels, facilitating clear identification of both the proximal and distal portions of the vein, and likely reducing the risk of vessel injury. In deep posterior mediastinal regions where the surgical field is narrow and adjacent to the intercostal vessels, the confronting upside-down setting offers a more ergonomic operative angle and a direct downward view, enabling secure dissection and precise vascular control.

This configuration allows the surgeon to approach the lesion from a higher, frontal perspective, thereby minimizing blind areas behind the vertebral column and improving spatial orientation.

Consequently, this approach may be particularly advantageous for tumors located along the vertebral bodies or in proximity to the major vessels. Furthermore, it is not limited to posterior mediastinal lesions and can also be effectively applied to those in the superior mediastinum [18] and supra-diaphragmatic regions [19].

Taken together, VATS using the confronting upside-down monitor setting is a feasible and effective option for the resection of posterior mediastinal lesions. Particularly in cases requiring vascular control or dissection near critical structures, this technique offers enhanced visualization and surgical operability.

## Conclusions

Although posterior mediastinal Müllerian duct cysts are extremely rare, they should be included in the differential diagnosis of posterior mediastinal cystic lesions, particularly in perimenopausal women. These cysts exhibit benign clinical behavior, and the prognosis after complete surgical resection is typically good. In particular, estrogen is thought to contribute to cyst development. Associated risk factors include obesity and gynecologic history, such as hormone replacement therapy. A definitive diagnosis requires a high index of suspicion and comprehensive histopathological evaluation, specifically immunohistochemical staining to prevent misdiagnosis. From a surgical standpoint, VATS using the confronting upside-down monitor setting provides excellent visualization with minimal instrument interference, thereby representing a valuable approach for the safe and effective resection of posterior mediastinal Müllerian duct cysts.
